# PPARG Binding Landscapes in Macrophages Suggest a Genome-Wide Contribution of PU.1 to Divergent PPARG Binding in Human and Mouse

**DOI:** 10.1371/journal.pone.0048102

**Published:** 2012-10-31

**Authors:** Sebastian Pott, Nima K. Kamrani, Guillaume Bourque, Sven Pettersson, Edison T. Liu

**Affiliations:** 1 Cancer Biology and Pharmacology 2, Genome Institute of Singapore, Singapore, Singapore; 2 Department of Microbiology, Tumor and Cell Biology, Karolinska Institutet, Stockholm, Sweden; 3 Computational and Systems Biology, Genome Institute of Singapore, Singapore, Singapore; 4 National Cancer Centre, Singapore, Singapore; National Institutes of Health, United States of America

## Abstract

**Background:**

Genome-wide comparisons of transcription factor binding sites in different species can be used to evaluate evolutionary constraints that shape gene regulatory circuits and to understand how the interaction between transcription factors shapes their binding landscapes over evolution.

**Results:**

We have compared the PPARG binding landscapes in macrophages to investigate the evolutionary impact on PPARG binding diversity in mouse and humans for this important nuclear receptor. Of note, only 5% of the PPARG binding sites were shared between the two species. In contrast, at the gene level, PPARG target genes conserved between both species constitute more than 30% of the target genes regulated by PPARG ligand in human macrophages. Moreover, the majority of all PPARG binding sites (55–60%) in macrophages show co-occupancy of the lineage-specification factor PU.1 in both species. Exploring the evolutionary dynamics of PPARG binding sites, we observed that PU.1 co-binding to PPARG sites appears to be important for possible PPARG ancestral functions such as lipid metabolism. Thus we speculate that PU.1 may have guided utilization of these species-specific PPARG conserved binding sites in macrophages during evolution.

**Conclusions:**

We propose a model in which PU.1 sites may have served as “anchor” loci for the formation of new and functionally relevant PPARG binding sites throughout evolution. As PU.1 is an essential factor in macrophage biology, such an evolutionary mechanism would allow for the establishment of relevant PPARG regulatory modules in a PU.1-dependent manner and yet permit for nuanced regulatory changes in individual species.

## Introduction

Evolutionary conservation is often used as a metric to estimate the biological significance of molecular components. This concept is embedded in the *in silico* annotation of DNA sequences where it is assumed that regions that are evolutionary conserved are more important than those that are not [1], [2]. However, recent studies that compared experimentally determined transcription factor (TF) binding sites [3], [4], [5], [6] have provided surprising evidence of a sizable divergence in binding events between species. Indeed, highly conserved regions appear to account for only a very small proportion of the total number of genome-wide binding sites. The low specificity of most DNA binding motifs, combined with the relaxed constraint on the binding position of regulatory proteins, can potentially allow for high plasticity in the binding site landscape between species. The high degree of observed species-specific binding is likely due to neutrally evolving sequences rather than the result of selective pressure [4] or changes in the binding specificity of the involved TFs [7]. The evolutionary gain and loss of binding sites is known as turnover and it has been reported to occur in mammalian regulatory networks [8]. However, to date, the forces acting upon and the dynamics of binding site turnover during evolution have only been explored in few experimental systems on a genome-wide scale [4], [5] and are generally not well understood. Additionally, the functional implications of this turnover on the underlying gene regulatory networks have not been addressed systematically.

To study the mechanisms that affect binding site turnover and to contribute novel insights into the evolutionary dynamics of transcriptional control in a mammalian system, we investigated the binding landscape of the nuclear receptor peroxisome proliferator-activated receptor gamma (PPARG) in human and mouse macrophages. PPARG is an essential regulator of adipogenesis [9], [10] and plays an important role in glucose homeostasis and inflammation. Upon ligand-activation PPARG heterodimerizes with one of the retinoid X receptors (RXRA, RXRB and RXRG, here collectively referred to as RXR) and binds to specific response elements (PPRE) [11]. In addition to the effects of PPARG in adipocytes [9], PPARG activity has been described in a variety of cell types and tissues [12], [13], [14]. In macrophages PPARG is also involved in the control of cholesterol metabolism and low or absent PPARG expression is associated with increased atherosclerosis [15], [16], [17] and insulin resistance [18], [19].

We, and others, have previously identified PPARG binding sites on a genome-wide level in mouse adipocytes [20], [21], [22], [23]. These data were complemented by genome-wide PPARG binding data in murine macrophages (Lefterova et al. 2010). In the mouse, PPARG binding profiles showed striking differences between macrophages and adipocytes and suggested a tissue-specific mechanism for binding site selection through additional TFs (i.e. PU.1 and CEBPs) [24]. As there are limitations in comparing two cell-types of different origins between two species, we obtained concordant data for a human macrophage cell line (THP-1) since it represents a well-characterized somatic cell type [25]. Here we report a genome-wide localization analysis for PPARG, RXR, and PU.1 in human macrophages and present a comprehensive interspecies analysis of PPARG binding sites and target genes in human and mouse macrophages.

## Results

### Genome-wide identification of PPARG/RXR binding sites in human macrophages

**Figure 1:**
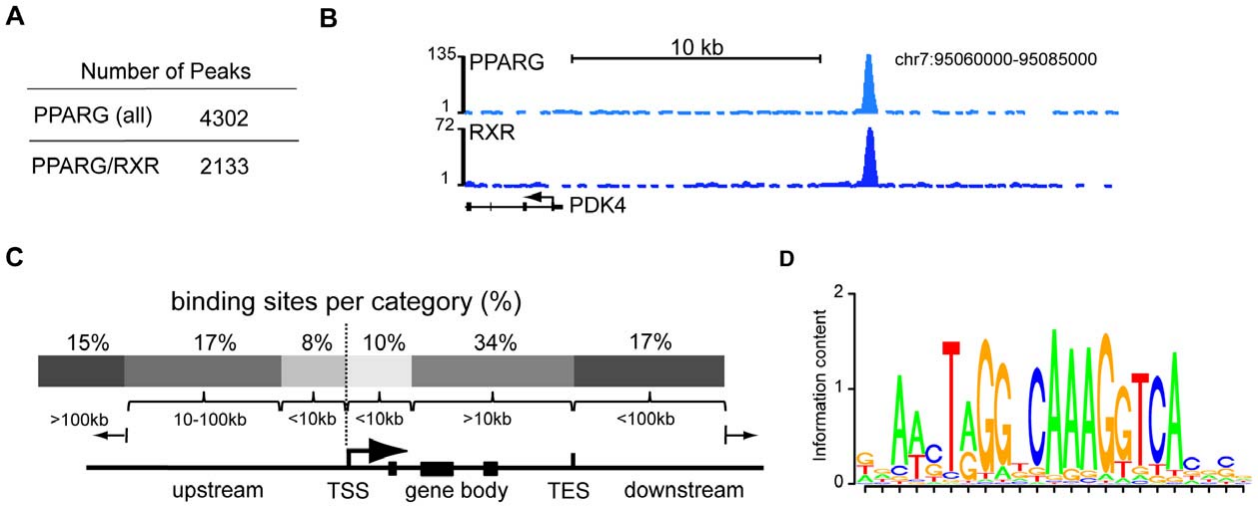
Global identification of PPARG and RXR binding sites in human macrophages. A) Table displaying the number identified PPARG peaks. PPARG/RXR peaks represent PPARG peaks that are supported by enrichment in the RXR ChIP-Seq library (RXR here represents RXRA, RXRB and RXRG). B) PPARG and RXR binding profiles across the locus for PDK4 in THP-1 cells. Plotted are the tag counts obtained from the respective ChIP-Seq libraries. C) Distribution of PPARG/RXR binding sites relative to annotated genes obtained from UCSC Genome Browser (built hg18/NCBI36; RefGene table). D) Motif identified *de novo* at PPARG/RXR binding sites using CisFinder.

The human monocytic cell line THP-1 [25] was used as a model to identify PPARG binding sites in macrophages. THP-1 cells express low levels of PPARG protein in the basal state, which increases substantially after treatment with PMA and during the subsequent differentiation (Fig. S1A). Treatment of differentiated THP-1 cells with the PPARG ligand Rosiglitazone (RSG) induced expression of known PPARG target genes. Chromatin-immunoprecipitation (ChIP) with antibodies against PPARG and its heterodimerization partner RXR enriches for PPARG/RXR binding loci in proximity to these target genes (Fig. S1B and C). Performing ChIP-seq for PPARg in PMA induced THP-1 cells we obtained a total of 4302 PPARG binding using the peak caller CCAT [26] (Fig. 1A; Table S1 and S2) (Material and Methods). To minimize the false-discovery rate we took advantage of the prerequisite for PPARG to interact with RXR in order to bind DNA [11], [27]. We obtained an additional ChIP-seq library for RXR, which served as independent biological replicate. PPARG peaks were only retained if they were additionally supported by RXR enrichment (see Material and Methods). The combination of PPARG and RXR binding data yielded a set of 2133 high confidence sites (Fig. 1A). These PPARG/RXR sites showed significantly stronger enrichment compared to PPARG sites without RXR binding (Fig. S1D). To rule out biases introduced by the peak calling algorithms we used a second peak caller (MACS, Zhang et al. 2008), to obtain PPARG and RXR peaks. The results of both algorithms are in good agreement (>84%), with most of the deviation observed for peaks with lower enrichment (Fig. S1E and F).

We find that PPARG/RXR binding sites occur throughout the genome but are enriched in proximity to genes, especially around the transcriptional start sites (TSS) with 18% of PPARG/RXR sites being located within 10 kb of the TSS (Fig. 1C). Further analysis of the identified PPARG/RXR binding regions identified de novo an enriched sequence motif that closely resembles the known PPARG recognition motif (Fig. 1D, Fig. S1G and H).

### Retention of PPARG binding in human and mouse macrophages is exceedingly low

**Figure 2:**
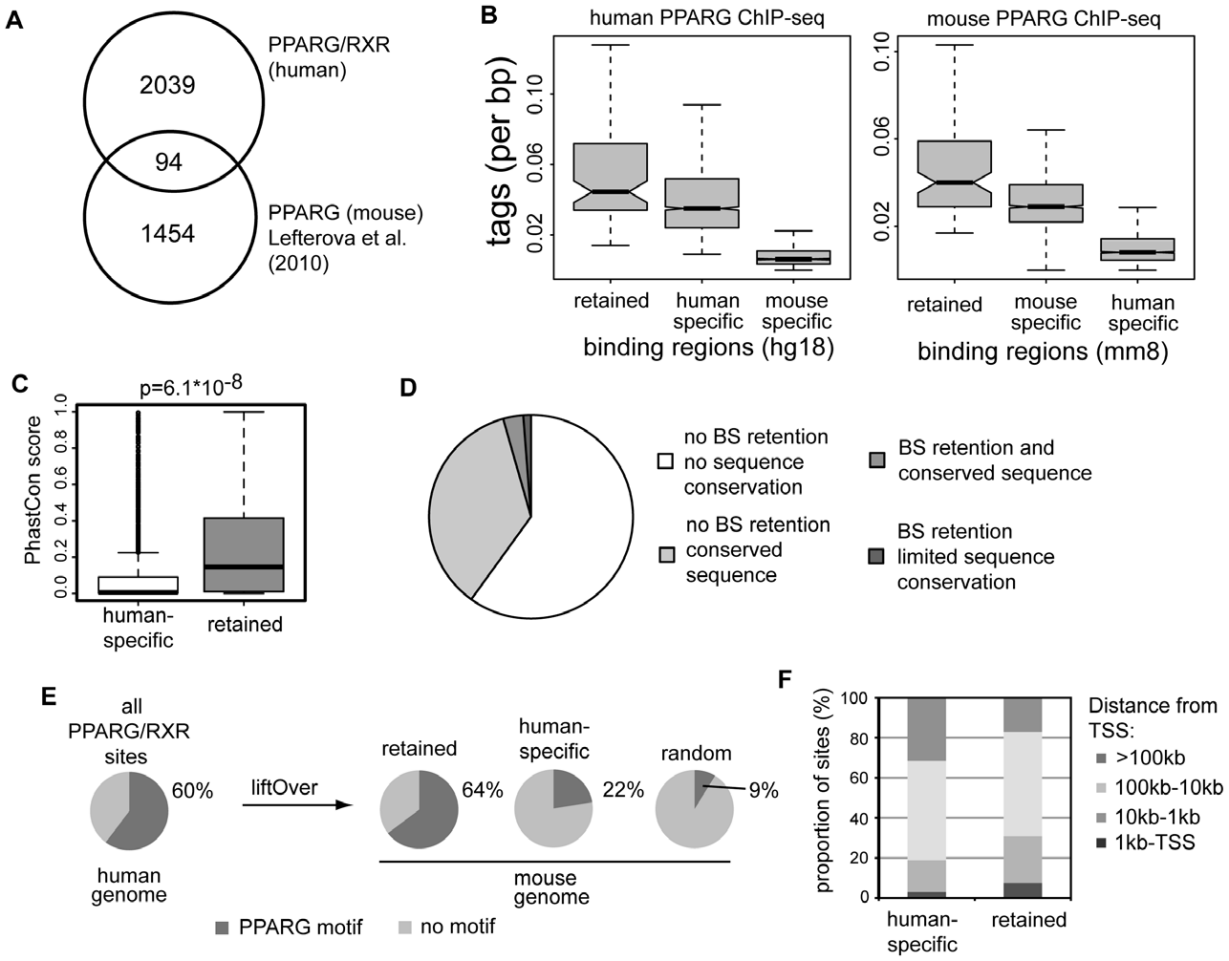
PPARG binding is poorly conserved between human and mouse macrophages. A) Overlap of PPARG bindings sites between human and mouse macrophages. Comparison is based on murine binding sites lifted over to the human genome. 1548 out of 1961 PPARG binding sites in mouse aligned to the human genome. B) Tag counts from human PPARG library at different genomic loci in the human genome and mouse genome. Retained binding sites, human-specific sites and mouse-specific binding sites. Mouse and Human-specific sites in the human and mouse genome refer to the orthologous loci of mouse-specific or human-specific sites in the original genomes. For better visualization outliers were omitted from plot. C) Sequence conservation at human-specific and retained PPARG/RXR sites. Shown is the distribution of PhastCons scores for both categories. Significance was calculated using two-tailed t-test D) Pie chart summarizing the proportion of PPARG/RXR site that are retained and/or show sequence conservation (i.e. overlap with PhastCons element). E) Proportion of PPARG/RXR sites in human macrophages containing a PPARG motif compared to the proportion of sites with motif after liftOver to the mouse genome. The orthologous regions in the mouse genome are separated into PPARG bound and not bound. ‘Random’ shows the expected motif frequency for randomly distributed intervals with a matched size distribution. F) Distribution of PPARG/RXR binding sites in regard to TSS of RefGenes. Displayed are the distributions of human-specific and conserved PPARG/RXR sites.

Recent studies reported limited overlap of transcription factor binding sites across species in several tissues [3], [4], [6]. Similarly, when we aligned the binding sites for PPARG in human macrophages against the published PPARG binding sites from mouse macrophages [24](Fig. S2), we only observed about 5% (94/2133) overlap between the two species (Fig. 2A;Fig. S2). These data suggest a massive change in the binding landscape through mammalian evolution. To avoid ambiguity in the term ‘binding site conservation’, i.e. between the conservation at the level of DNA sequence and ‘physical’ conservation where binding is observed in both species at orthologous loci, we refer to the inter-species overlap of empirically determined binding sites as ‘retention’ similar to Schmidt et al. [28]. A potential pitfall of such inter-species comparison is the fact that peak-calling programs detect peaks above a certain threshold, thus transforming the continuous distribution of different peak heights into a binary signal. Because of this, it is possible that a fraction of enriched regions that had not achieved the threshold value were discarded and this might led to false-negatives (i.e. retained sites that were falsely labeled human-specific). To address the potential impact of this effect we compared the tag counts in human and mouse ChIP-seq libraries at retained binding sites, human-specific, and mouse-specific sites for both the human and mouse PPARG ChIP-seq libraries. In the case of many false-negative peak calls due to threshold effects one would expect to see significant PPARG binding at supposedly mouse-specific loci and vice versa. However, the comparison of tag counts between the different binding regions revealed virtually no enrichment at the mouse-specific loci in humans and vice versa (Fig. 2B). In addition, using sets of binding sites obtained under different significant thresholds has only marginal effects on the proportion of retained sites and even under the most stringent peak calling conditions the proportion of retained binding sites did not approach 10% (Fig. S2E). Of note, retained binding sites show generally higher tag counts than species-specific binding sites (Fig. 2B, Fig. S2C).

The strong divergence in PPARG binding prompted us to address the extent of sequence conservation at retained binding sites. We found that, on average, retained binding sites also showed significantly greater sequence conservation compared to binding sites that were not retained in mice (p = 6.1*10^−08^, comparison based on an aggregated score of multi-species alignment)(Fig. 2C). We therefore asked whether such regional sequence conservation alone was sufficient to explain retention of PPARG binding sites or whether additional determinants might play a role. To test this, we assessed the number of human PPARG/RXR binding sites in macrophages that showed some degree of sequence conservation; regional conservation was inferred from overlap with PhastCons elements [29] (See Material and Methods). In total 40% of all PPARG/RXR binding sites in human macrophages overlapped a PhastCons element (Fig. 2D) and therefore showed some degree of sequence conservation. However, regional sequence conservation alone was not a strong predictor of binding site retention since only 8% of these sites were found to be also bound in mice.

Isolated PPRE can drive PPARG binding and we therefore asked whether the presence of a recognizable PPARG/RXR motif is required to discern PPARG binding within orthologous regions. For a direct comparison the human binding regions were lifted-over to the mouse genome to identify orthologous segments in the mouse. We detected the PPARG/RXR motif in 60% of human binding peaks. A comparable proportion of PPARG/RXR motifs (64%) were found within the retained PPARG binding regions in mouse (Fig. 2E). However, human-specific binding sites showed a significant reduction in PPARG/RXR motif occurrence (22%) at the orthologous loci in mice (Fig. 2E). Motif scanning using various cut-offs suggests that most of the binding regions (up to 90%) harbor sequences that match to the PPARG motif (Fig. S2F). To maintain an acceptable false-positive rate we decided to use a more conservative estimate. This suggests that regional sequence conservation alone cannot explain the retention of binding sites between the two species and that the presence of the binding motif is a major driver for PPARG binding. The absence of the PPARG binding motif at non-retained sites provides evidence that the observed differences in binding between human and mouse are caused by genetic differences (i.e. presence or absence of motif) rather than epigenetic differences (e.g. because of subtle differences in the compared cell types between human and mouse).

Furthermore, we found that the genome-wide distribution of retained PPARG/RXR binding site differed from human-specific sites. Retained sites were preferentially located in the proximity to genes (≤10 kb) (Fig. 2F): 30% (29/94) of retained sites are found within 10 kb of TSS compared to less than 20% (385/2039) of human-specific sites. Conversely, more than 30% (642/2039) of the human-specific sites are located more 100 kb away from the TSS of genes, with only 17% (16/94) of retained binding sites are located distally.

### PPARG binding in human and mouse delineates species-specific and shared target genes

**Figure 3:**
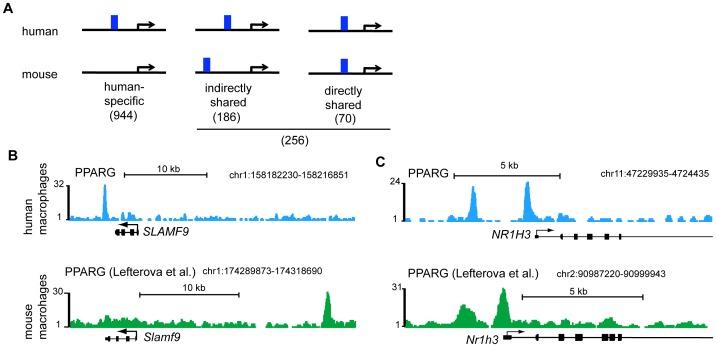
Identification of human-specific and shared PPARG/RXR target genes. A) Grouping of PPARG/RXR targets genes in human macrophages based on PPARG binding in mouse. Displayed is the number of genes that are human-specific, indirectly, and directly shared PPARG/RXR target genes. Only PPARG binding sites in proximity to genes (<100 kb to TSS) were taken into consideration. B) and C) Enrichment of PPARG binding in homologous regions proximal to *SLAMF9/Slamf9* and *NR1H3/Nr1h3* in human and mouse macrophages (upper and lower panel, respectively). *SLAMF9/Slamf9* represents an indirectly shared PPARG target gene while *NR1H3/Nr1h3* represents a directly shared target gene. Browser tracks for mouse are shown in reversed direction to facilitate easier comparison between human and mouse.

The vast majority of human PPARG binding sites were not retained in mouse macrophages. Furthermore, retained and human-specific bindings sites differed in several aspects (e.g. binding site enrichment, genomic location). We therefore asked if and how differences between retained and human-specific binding sites might relate to gene regulation. Several studies have demonstrated that regulatory control of a target gene by a specific TF can be maintained during evolution in the absence of a retained binding site. It has been shown that the emergence of novel TF binding sites in the vicinity of the regulated gene can compensate for the loss. Such binding site turnover has been demonstrated for different factors [3], [4]. Therefore, species-specific loss or gain of PPARG binding sites might be compensated for by the emergence of novel sites compared to the ancestral state. To assess this kind of binding site turnover we first defined putative PPARG target genes as genes with at least one PPARG binding site within 100 kb of the TSS (Table S3; See Material and Methods). We then grouped these genes into human-specific targets if binding only occurred in humans but not mouse and shared target genes if PPARG binding sites were observed in both human and mouse. Shared target genes may be associated with retained PPARG binding sites or with divergent binding sites that reside at distinct genomic segments in the two species but within 100 kb of the TSS of a common target gene. We therefore separated shared target genes into directly shared (i.e., genes adjacent to retained binding sites) and indirectly shared target genes (i.e. genes adjacent only to loci that are species-specific binding sites) (Fig. 3A and Table S4). Out of 1200 PPARG/RXR target genes identified in human macrophages, 944 were specific to human while 256 genes (21%) were shared between human and mouse macrophages (Fig. 3A). Out of the 256 shared targets 186 (73%) were indirectly shared and 70 genes (27%) were associated with retained binding sites and therefore represented directly shared targets (Fig. 3A). These data show that the majority (4/5) of the putative target genes in human macrophages appear to be specific to humans, and that the majority (3/4) of the shared target genes of PPARG are not in proximity to conserved binding segments in human and mouse. For example, *SLAMF9* which exhibits divergent PPARG binding with a binding site that is located downstream of the TSS in human macrophages while it is located upstream of *Slamf9* in the mouse (Fig. 3B). By contrast, *NR1H3/Nr1h3*, a directly shared target gene, shows retained PPARG binding in human and mouse macrophages (Fig. 3C).

### Retained PPARG binding sites are enriched at target genes induced by PPARG ligand

**Figure 4:**
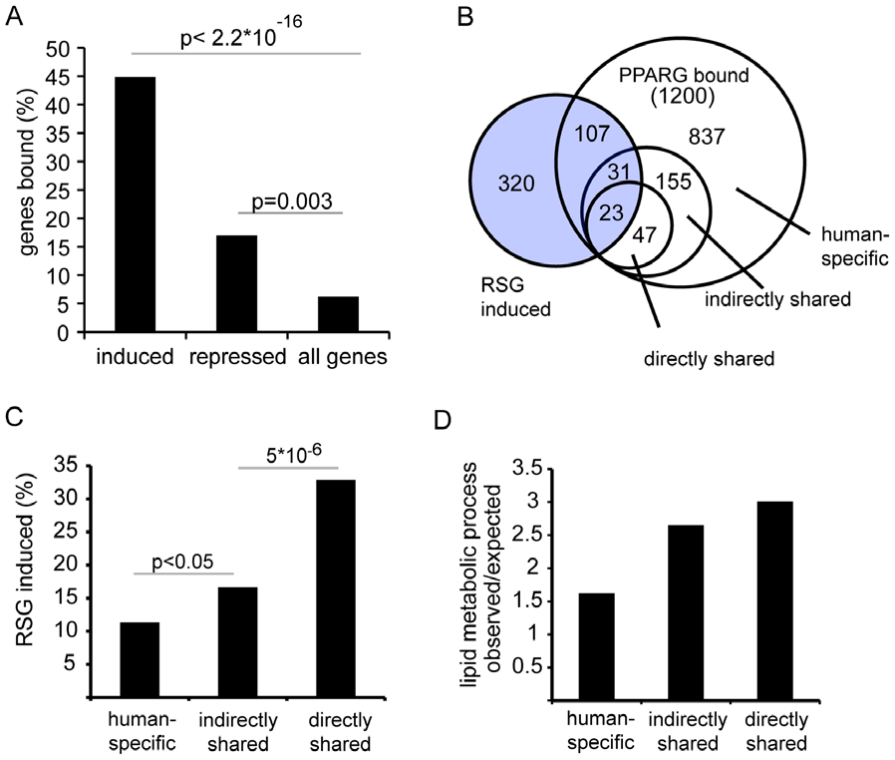
Conservation reveals functional PPARG/RXR target genes. A) Association of PPARG/RXR binding sites with RSG regulated genes in THP-1 cells. Significance of enrichment over background was calculated using Fisher's exact test. B) Venn diagram representing the overlap between PPARG/RXR bound genes and RSG regulated genes across the different conservation categories. Indicated are the numbers of genes exclusive to the respective gene sets. C) Proportion of non-conserved, indirectly and directly shared target genes that are induced by RSG. Significance was calculated using Fisher's exact test. D) Bar plot showing the ratio of expected versus observed number of genes associated with the biological process category ‘lipid metabolic processes’ obtained from PANTHER for human-specific, indirectly and directly shared target genes.

The three categories of PPARG putative target genes (human-specific, indirectly and directly shared) were purely defined on the basis of PPARG binding, we therefore asked if genes within these categories differ in the response to PPARG ligand. To this end, we identified Rosiglitazone (RSG)-responsive genes in THP-1 cells by genome-wide expression analysis. As expected, the correlation of RSG-responsive genes with PPARG binding sites revealed a strong overrepresentation of direct PPARG targets among RSG induced genes in general (Fig. 4A and Table S5). This association was confirmed using a second set of RSG-responsive genes generated in a related myeloid cell type, human dendritic cells [30](Fig. S3A–C). We pooled the two gene sets to increase the sensitivity for detection, yielding a total of 481 RSG-responsive genes, and compared this list to the previously annotated sets of human-specific, indirectly and directly shared PPARG/RXR targets. This analysis identified 161 PPARG/RXR target genes that were also regulated by RSG in human macrophages. Notably, one-third (54) of these were shared PPARG targets genes of which 31 were shared indirectly and 23 were directly shared targets (Fig. 4B and Table S6). Hence, target genes adjacent to retained PPARG binding sites were about 3 times more likely to be regulated by RSG than human specific target genes (33% vs. 11%), while indirectly shared target genes were only 1.5 fold more likely to be regulated than human-specific targets (17% vs. 11%; p<0.05)(Fig. 4C). In line with this observation, we found a significant enrichment of genes associated with the functional term ‘lipid metabolic process’ for both categories of shared target genes as compared to human-specific target genes (Fig. 4D, Fig. S3D and E). These results are supported by expression data from murine monocytes deficient in *Pparg* [18] which reproduced a similar correlation with the three groups of target genes (mouse-specific, indirectly and directly shared target genes) showing progressively greater fractions of differentially regulated genes (Fig. S3F).

These data suggest that a limited set of core PPARG/RXR target genes, associated with retained, sequence conserved binding sites, may represent the primordial function of PPARG in macrophages. This function seems to be primarily associated with lipid metabolism.

### The macrophage-specific configuration of cis-regulatory modules is conserved

PPARG binding in murine macrophages correlated strongly with binding sites for PU.1 and suggested that establishment of tissue-specific binding sites was in part dependent on PU.1 [24]. Consistent with the results in mouse macrophages, we found an enrichment of a DNA sequence motif for ETS family factors within human PPARG/RXR binding sites. (Fig. S4A–C). Of note, enrichment of ETS motifs was specific to PPARG binding sites in human macrophages. The proportion of sites with and ETS motif was less than half of that in macrophages when scanning PPARG binding sites obtained in human adipocytes (39% vs. 17%) (Fig. S4D and E).

**Figure 5:**
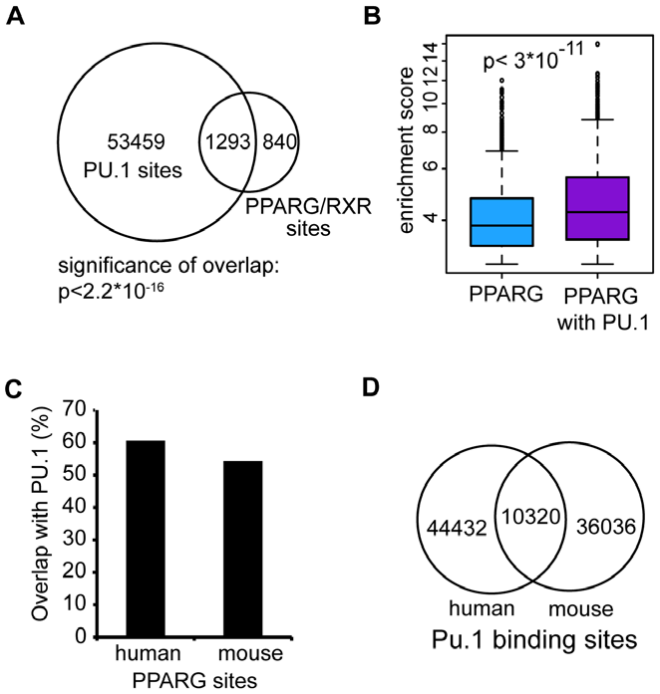
Composition of PPARG bound cis-regulatory modules is conserved between human and mouse macrophages. A) Overlap between PPARG/RXR and PU.1 ChIP-seq peaks. Significance of overlap was calculated using proportion test. B) PPARG/RXR ChIP-Seq enrichment at PPARG/RXR sites without and with PU.1 overlap. C) Proportion of PPARG/RXR binding sites in human and mouse macrophages that are co-occupied by PU.1. D) Venn-diagram depicting the numbers of species-specific and retained PU.1 binding sites in human and mouse macrophages.

Given that we found ETS motif enriched in human PPARG peaks and notwithstanding the limited retention of PPARG binding between human and mouse, we asked whether PU.1 binding at PPARG sites was also important in human macrophages. To address this question directly we generated a PU.1 ChIP-Seq library and identified 54,752 PU.1 binding sites in human macrophages (Table S7). The number of PU.1 binding sites found in human macrophages is comparable to that of PU.1 binding sites identified in mouse (46,356) [24] and we found that 60% (1293/2133; p<2.2*10^−16^) of the human PPARG/RXR binding sites were co-occupied by PU.1 (Fig. 5A, Fig. S4G–I). In addition, the level of PPARG occupancy at sites shared with PU.1 was significantly greater than at PPARG/RXR sites without PU.1 (P<3*10^−11^, Fig. 5B). These data suggest that PU.1 has an augmenting effect on PPARG binding, and that this co-occupancy is driven in large part by juxtaposition of cognate DNA recognition motifs. Intriguingly, despite the very low retention of individual PPARG binding sites between human and mouse (see Fig. 2A), the co-occurrence of PPARG and PU.1 binding in the genome is found at equally high frequency in human and mouse macrophages (∼50–60%, Fig. 5C). It is of note that we found the retention of PU.1 binding sites to be higher than for PPARG/RXR binding (approximately 19% vs. 5%) (Fig. 5D).

Based on the observation that PU.1 co-binding at PPARG binding sites was frequently observed in both species, we asked whether PU.1 could act as an additional determinant for PPARG binding at conserved PPARG sites. To test this, we selected human PPARG/RXR binding sites that contained a PPRE at orthologous loci in both species. These PPARG/RXR sites were then split into retained and human-specific sites. Retained PPARG/RXR sites showed a high proportion of PU.1 co-binding and the presence of a PU.1 motif at the both the human and mouse loci. Conversely, PU.1 binding and motif occurrence were significantly reduced at mouse loci corresponding to human-specific PPARG/RXR sites (Fig. S5), correlating with the loss of PPARG/RXR binding. These data suggest that PU.1 acts as a determinant for PPARG binding in the evolutionary context and that this co-occurrence is more common in retained sites.

### Binding site turnover might be facilitated by regulatory modules

**Figure 6:**
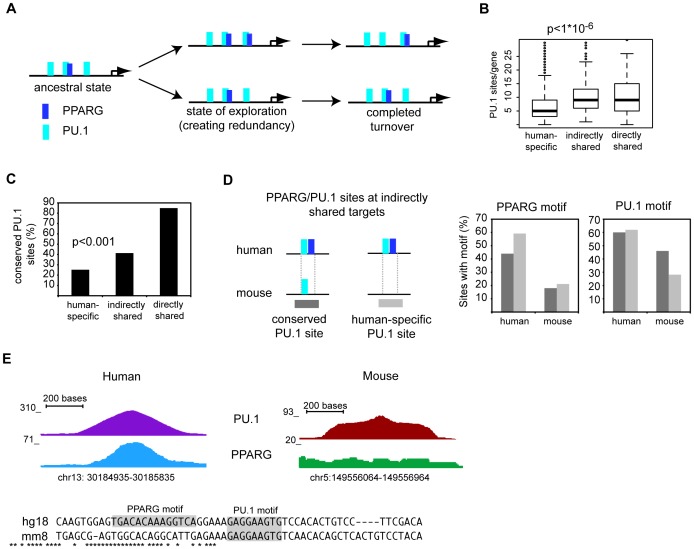
Pu.1 potentially restricts binding site selection for PPARG during binding site turnover. A) Scheme depicting a potential scenario for PU.1-associated PPARG binding site turnover. B) Average numbers of PU.1 binding sites in proximity to human-specific, indirectly shared, and directly shared PPARG target genes (<100 kb of TSS). Significance was calculated using two-tailed t-test. C) Proportion of conserved PU.1 binding sites at PPARG/RXR-PU.1 binding sites in human macrophages. Comparison was made between sites at human-specific and indirectly shared targets and significance was calculated using Fisher's exact test D) Human PPARG/RXR binding sites co-bound by PU.1 and adjacent to indirectly shared genes were split into sites containing conserved PU.1 binding sites and human-specific PU.1 binding sites, respectively. PPARG and PU.1 motifs were identified at orthologous loci in human and mouse. E) Shown is the locus for a PPARG/RXR binding site in human macrophages adjacent to *ALOX5AP* and its orthologous region in mouse. Binding for PU.1 and PPARG is shown at orthologous regions in human and mouse. Sequence alignments demonstrate conservation and loss/gain of binding motifs.

Two mechanisms for evolutionary divergence in regulatory sites have been described by recent publications; neutral mutational drift [4], or use of transposable elements [3], [31]. While transposon-mediated dispersal of binding sites provides an attractive model for acquisition of TF-specific novel binding sites, we did not detected significant association of PPARG binding sites with specific families of transposable elements in either human and mouse macrophages (data not shown). Since transcription factors frequently cooperate at binding sites to increase DNA binding or to stabilize DNA binding thus potentiating transcription [22], [23], [32], [33], we asked whether DNA binding of the lineage-specific TF PU.1 could influence the selection of PPARG binding sites during evolution. In such a model, the PU.1 binding sites (∼54,000) would act as regional ‘anchors’, which restrict the recruitment of the PPARG protein to sites with PPRE sequence ‘seeds’ (Fig. 6A). This scenario would allow for binding site turnover yet restrict this evolutionary exploration to loci that are more likely to be functionally relevant in macrophages. In line with a potential role of PU.1 in the turnover of functional PPARG binding sites, we found that indirectly and directly shared target genes have a higher average number of PU.1 binding sites per gene compared to human-specific targets (Fig. 6B). Retained PPARG/RXR sites that were also occupied by PU.1 in human macrophages were assessed for PU.1 binding the mouse genome. By definition, these sites were bound by PPARG in mice. We found that 85% of these loci also contain retained PU.1 binding sites (Fig. 6C). This suggests a strong correlation between retained binding of PPARG and PU.1. We then hypothesized that PU.1 may act as an ‘anchor’ for PPARG binding in evolution and that the highly conserved sites that harbor both TFs in either species serve as the primordial regulatory collection. This model would suggest that PPARG/RXR binding sites at indirectly shared targets would contain fewer ancestral PU.1 binding sites than the retained PPARG/RXR sites, but in turn would have a greater proportion of ancestral PU.1 sites than found in human-specific target genes. In agreement with the model, whereas retained PPARG/RXR sites show 85% overlap with retained PU.1 sites, this was reduced to 41% in the PPARG/RXR-PU.1 sites adjacent to indirectly shared genes and followed lastly by only 25% of the PPARG/RXR-PU.1 sites at human-specific targets (p<0.001) (Fig. 6C).

We then asked if the discrepancies in the physical PPARG and PU.1 binding between mouse and human were a result of losses or gains of the cognate motifs for the co-occupying TFs. We examined the proportion of PPARG and PU.1 motifs at human PPARG/RXR-PU.1 co-binding loci near indirectly shared genes both in the human and in the orthologous regions in mouse. The PPARG/RXR-PU.1 sites were split into two groups, one containing PPARG/RXR binding sites that were co-occupied by a retained PU.1 site while the sites in the other group were co-occupied by human-specific PU.1 binding sites (Fig. 6D). We found, in both circumstances, that the PPARG/RXR motif was lost at the non-bound orthologous position in the mouse. Furthermore, retained PU.1 sites showed a higher proportion of PU.1 motifs in mouse as compared to the murine loci corresponding to human-specific PU.1 sites. This implicates motif conversion as a major cause of binding site turnover for both PPARG/RXR and PU.1. In one example, the PPARG binding locus in proximity to *ALOX5AP/Alox5ap*, an indirectly shared target, showed physical PU.1 binding and the presence of a PU.1 motif both in human and mouse while selective PPARG binding in humans is associated with a human-specific PPARG motif at this locus (Fig. 6G). A more elaborate example is provided by the LIPA/Lipa locus (Fig. S5B, C). Together, these examples would be in agreement with a model in which a part of evolutionary new binding sites for PPARG would be established at pre-existing binding loci of PU.1.

## Discussion

We provide a genome-wide interspecies analysis of PPARG and PU.1 binding locations in human and mouse macrophages. Our analysis revealed a low degree of PPARG binding site retention (∼5%), which did not significantly increase when including only very strong binding sites (Fig. S2). Despite such limited binding site retention, functional target genes of PPARG are strongly enriched for binding in both species. Our results reveal a gradient of regulatory control of PPARG targets associated with the different types of adjacent PPARG binding sites: directly shared target genes (i.e. retained binding sites adjacent to responsive genes) are most tightly associated with PPARG-dependent gene regulation followed by indirectly shared targets (i.e. non-overlapping binding sites in the two species but adjacent to the same target gene), while human-specific target genes are more loosely associated (Fig. 4). Furthermore, the hematopoietic lineage-specification factor PU.1 co-occupies the majority of PPARG binding sites in human and mouse macrophages in a similar manner (Fig. 5C), which supports the role of PU.1 as a major determinant for PPARG binding in myeloid cells.

Combining the analysis of these experimentally determined PPARG and PU.1 binding sites, we propose that PU.1 might contribute to PPARG binding site turnover during evolution. This model incorporates genomic data suggesting that PPARG binding is enhanced by the presence of PU.1 (Fig. 5B). PU.1 is required for the specification of the myeloid lineage [34] and crucial for the establishment of open chromatin regions and functional enhancers in mouse macrophages [35], [36]. Therefore, exploration towards functional PPARG binding sites could be facilitated as PU.1 might act as ‘anchor’ for PPARG at nascent, low-affinity PPREs located within active macrophage enhancers. In the absence of PU.1 binding these sites would not be accessible to PPARG/RXR. We think that this model represents the logical extension of the role of PU.1 in determining binding site accessibility. This model predicts that functional new PPARG sites resulting from evolutionary turnover should be skewed towards PU.1-dependent enhancer regions already established in the ancestral state. Indeed, we found that the PU.1 binding site within PPARG-PU.1 binding loci was more likely retained at indirectly shared PPARG target genes than it was at species-specific PPARG target genes (Fig. 6C). A consequence of this form of PU.1-associated binding site turnover is that it would permit the exploration of new and adaptive regulatory solutions for this important nuclear hormone receptor in a ‘guided’ rather than fully random manner since PPARG would ‘co-opt’ already existing regulatory modules and enhancers.

Dramatic changes in TF binding at orthologous loci across species have been observed in previous studies [3], [6]. We, and others, have found association of species-specific binding site turnover for critical factors such as p53 and Oct4 with dispersal of retrotransposons and repetitive elements [3], [37], [38]. In studies of closely related drosophila species quantitative changes in TF binding at homologous loci have been in part attributed to factors not directly related to the TF binding sequence, such as nucleosome positioning and chromatin structure [5]. The findings from these inter-species comparisons are complemented by studies which demonstrate that single-nucleotide polymorphisms influence TF binding even if located outside of the primary binding motif, presumably by influencing binding of a cooperation partner in cis, and contribute to regulatory variation among human individuals and in yeast [39], [40], [41]. Thus, a common theme of these studies is a high degree of regulatory diversity. Here, our data further suggest that changes in the binding landscape of a particular TF during evolution might be strongly influenced by sequence mutations at binding sites near a second, collaborative TF. It is likely that these observations would not be restricted to PPARG alone but would be generally observed for TFs for which PU.1 acts as an additional lineage-specific determinant of binding site selection. It is of note that this mechanism of PU.1-associated PPARG binding site turnover is only one aspect of the evolutionary processes influencing PPARG binding. For example, PPARG activity is crucial in adipocyte biology, however PU.1 expression is absent in adipocytes and significant differences in PPARG binding between murine macrophages and adipocytes have been reported [24]. Of interest, however, is that despite these differences, the PPARG sites highly conserved between human and mouse macrophages are also enriched in sites bound in murine adipocytes (data not shown) suggesting an evolutionary conserved and tissue-independent function of PPARG, mediated through these conjoint PPARG/RXR-PU.1 binding sites. Almost all of these sites were bound by PU.1 both in human and mouse macrophages. Conserved PU.1 co-binding therefore appears to be essential for PPARG ancestral functions. Given the tissue-restricted expression of PU.1, we surmise that the primordial PPARG program must encompass primary macrophage-specific functions and that PU.1 is required for macrophage-specific functionalization of these sites.

More generally, cross-species comparison of TF binding data or enhancer marks might therefore provide a powerful approach to identify biologically important loci and gene targets. Indeed, by combining H3K27ac data from human and mouse during adipogenesis such a strategy led to the identification of novel regulators of adipogenesis [31]. Given that many TFs bind cooperatively, a combinatorial conservation analysis of groups of such interdependent TFs might therefore facilitate a better understanding of the dynamics that shape gene regulatory networks during evolution and provide a higher order view of function conservation.

## Methods

### Cell culture

THP-1 cells were obtained from ATCC and maintained at >2×10∧5 cells/ml in RPMI 1640 medium supplemented with 10% FBS (Gibco) and Penicillin and Streptomycin (Gibco).

### Chromatin immunoprecipitation

THP-1 cells were activated with phorbol myristate acetate (PMA) (50 ng/ml)(Sigma) for 24 h to obtain cells with macrophage-like characteristics and treated with 1 µM Rosiglitazone (RSG) (Cayman Chemical) for 1.5 h before harvesting. Cells were cross-linking with 1% formaldehyde for 10 minutes. Excess formaldehyde was quenched by addition of glycine (0.625M). Cells were washed with cold PBS, trypsinized, and collected (3000 rpm for 15 min at 4°C; Sorvall Legend RT). Pellet was resuspended in Triton X lysis buffer (0.25% Triton X-100, 10 mM EDTA, 10 mM Tris.HCl[pH 8.1], 10 mM NaCl, 1X protease inhibitor) and incubated for 30 min. Nuclei were collected (3000 rpm for 15 min at 4 C; Sorvall Legend RT) and approximately 1*10∧7 nuclei where resuspended in 300 ul SDS lysis buffer (1% SDS, 5 mM EDTA, 50 mM Tris.HCl[pH 8.1],1x protease inhibitor). Nuclei were lysed for 30 min after which sonication was used to fragment the chromatin to an average size of 200–500 bp. Cellular debris was removed by centrifugation at 136000 rpm at 4 C in table top centrifuge (Eppendorf). 300 ul of nuclear lysates were diluted 1∶10 with dilution buffer (1% Triton X-100, 2 mM EDTA, 20 mM Tris.HCl[pH 8.1],150 mM NaCl, 1X protease inhibitor), chromatin was pre-cleared with 250 ul of Protein A-Sepharose bead slurry (CL-4B, Invitrogen) for two hours. After pre-clearing, protein-DNA complexes were immuno-precipitated using 5 ug of mouse IgG, PPARG (PP-A3409A-00, PPMX), RXR(delta197) (sc-774X, Santa Cruz), PU.1 (sc-352, Santa Cruz), respectively, and 75 ul of sepharose-A beads overnight. The beads were washed and protein-DNA complexes were eluted with 150 ul of elution buffer(1% SDS, 10 mM EDTA, 50 mM Tris.HCl[pH 8.1]) subjected to protease treatment and de-crosslinked at 65°C overnight. After phenol/chloroform extraction DNA was isolated by ethanol precipitation.

### ChIP sequencing and peak calling

Libraries were prepared from 10 ng of purified ChIP DNA according to the manufacturer's protocol (Illumina). ChIP-seq data were generated using Illumina GA single-read sequencing. Sequenced tags were mapped to the human genome (hg18/NCBI36) using ELAND (Illumina), only uniquely mapped tags were retained. Regions enriched in the ChIP samples were identified using CCAT [26]. Significance of enrichment was calculated compared to the IgG control library, peaks with FDR<0.01 were used for further analysis. 4302 peaks with FDR<0.01 were identified for PPARG and 54752 k for PU.1. To identify RXR peaks that support PPARG we considered all peaks with a liberal threshold of >2 fold. 2133 peaks were identified as PPARG/RXR binding sites. In addition we used MACS (Model-based analysis of ChIP-seq) (Zhang et al. 2008) as a second peak caller. MACS was used with default parameters, with the Mfold parameter set to 16 and 10 for human and mouse ChIP-seq libraries, respectively. When using MACS to test the influence of thresholds on binding retention, the cut-off p-value was varied between 10^−7^ and 10^−4^.

Gene coordinates were obtained from UCSC RefGene table (NCBI36 hg18) and binding sites were mapped to the nearest gene (within 100 kb). For visualization of the binding profile at specific loci the density of sequenced tags were displayed on a UCSC browser track. To smoothen the profiles, tags were extended to 250 bp. We used the galaxy platform [42] (http://main.g2.bx.psu.edu/) and functionalities embedded in BEDtools [43] for analyzes based on binding site coordinates.

### Motif identification and enrichment

CisFinder [44] (http://lgsun.grc.nia.nih.gov/CisFinder/) was used for the identification of sequence motifs enriched in the 150 bp regions surrounding the center of each PPARG/RXR binding peak. CisFinder was run with default settings and the highest ranked motif clusters were selected for further analysis. Motifs identified with CisFinder were matched against published motifs using STAMP [45] (http://www.benoslab.pitt.edu/stamp/) with default settings. For scanning of motif frequency, the search motif function in CisFinder was run on 200 bp sequences containing the binding sites on the human genome and on the homologous locations after lift-over.

Analysis for motif enrichment was complemented using Meme-ChIP (http://meme.sdsc.edu/meme/intro.html) (Machanick et al. 2011). Briefly, using MEME we obtained a PPARG motif nearly identical to the one found with CisFinder. This motif was then used to scan all binding site for its presence using FIMO (Grant et al. 2011) with default settings.

### Enrichment of biological processes and pathways

Biological processes and pathways enriched among PPARG/RXR target genes were identified using the Panther database [46] (http://www.pantherdb.org). Enrichment of biological processes specifically associated with putative PPARG/RXR targets was calculated by comparison to the expected proportion of genes associated with the respective process in a background of all human genes.

### Interspecies comparison

Binding sites in human and mouse were compared by obtaining the orthologous regions of the published mouse PPARG (total 1961) and PU.1 (total 46356) binding sites [24] in the human genome using the liftOver function in Galaxy (from mm8 to hg18) [42], [47]. To ensure efficient lift-over we used 1 kb intervals with a minimum ratio of bases that must remap of at least 0.1. To get conservative estimates human PPARG binding regions were extended to 1 kb as well while for PU.1 the human regions were kept at 150 bp. Conservation of target genes was addressed by lifting the coordinates of human genes (txStart to txEnd) from the RefGene table (hg18) to the mouse genome (mm8) using liftOver with a minimum ratio of bases that must remap at least 0.1. Genes with at least one binding site within 100 kb of the TSS were considered targets.

### Sequence conservation of binding sites

Differences in sequence conservation between in vivo conserved and human-specific PPARG/RXR sites was assessed by using the average PhastCons (17-species multiz alignment) score for each region. To test if sequence conservation generally predicted in vivo conservation of human PPARG/RXR binding sites, genomic intervals encompassing the centre of each peak were overlapped with PhastCons elements. PhastCons scores and elements were identified through global alignments of several vertebrate genomes [29] and downloaded form UCSC Genome Browser [47].

### Expression analysis

Following treatment with PMA cells were washed with PBS and incubated with medium without PMA (as we found that this procedure increased RSG responsiveness). Per sample 1×10∧6 cells were seeded and treated with 1 uM RSG or vehicle (DMSO). Cells were harvested after 0.5 h, 1.5 h, 3 h, 8 h, 12 h, respectively, and we obtained 5–6 replicates per condition. Isolation of RNA was done using the RNeasy kit (Qiagen) following manufacturer's introductions. For microarray expression analysis cRNA was prepared from 750 ng of isolated mRNA using the Ambion cRNA kit. For each sample 500 ng of cRNA were hybridized to Illumina BeadChips-8 Version2 according to manufacturer's protocol. IlluminaBead Chip (Illumina) Chips were scanned and probe intensities were measured with Illumina Beadscan. Probe intensities were normalized using average normalization in BeadStudio. Differentially expressed genes were identified using a linear model based on treatment and time in R (lm module). Additionally data from Szatmari et al. [48] was used to increase the sensitivity. From this study we used all RSG induced genes from all timepoints (6 h and 12 h and 5 d) for the analysis.

### Additional information

MIAME compliant Illumina expression array data and sequencing data have been submitted to the NCBI Gene Expression Omnibus (GEO) database and are accessible as SuperSeries under the accession number GSE25608.

## Supporting Information

Figure S1 **PPARG ChIP-seq reliably detects PPARG binding sites in THP-1 cells.** A) Western blot for PPARG after and before PMA differentiation of THP-1 cells. KU70 was used as control. B) Induction of mRNA levels of *PDK4* and *ANGPTL4* in PMA differentiated THP-1 cells after RSG treatment. Shown is the fold induction compared to vehicle (DMSO) treated cells. RNA was harvested at indicated timepoints C) ChIP enrichment detected at PPARG binding sites adjacent to *PDK4* and *ANGPTL4* using antibodies against PPARG and RXR D) enrichment score determined by CCAT for PPARG peaks at PPARG binding sites without and with RXR support E) Comparison of peaks obtained with different peak calling algorithms. Venn-diagrams showing overlap between peaks called with CCAT and MACS, respectively. Left diagram shows PPARG peaks and PPARG/RXR peaks are compared to the right. F) Overlap between CCAT and MACS calls for PPARG/RXR peaks for quartiles of PPARG/RXR binding sites. Quartiles are based on CCAT enrichment score. The proportion of PPARG/RXR peaks called with CCAT that overlaps a PPARG/RXR peak called with MACS is shown for each quartile G) Transfac motif matching the identified PPARG/RXR binding motif; Similarity calculated with STAMP H) Identified PPARG/RXR motif is detected at the majority of binding sites and located at the centre of the peak (300 bp interval).
(TIF)

Figure S2 **Low binding site retention is robust to different peak calling thresholds.** A) Comparison of PPARG peaks in murine macrophages reported by Lefterova et al. and peaks called from the same raw data using MACS. B) Overlap between original peaks published by Lefterova et al. and MACS calls for PPARG. Peaks called by Lefterova et al. were split into quartiles based on reported peaks scores. The proportion of PPARG peaks in the original dataset that overlaps a PPARG peak called by MACS is shown for each quartile. C) Binding site retention is biased towards strong PPARG/RXR peaks. Human PPARG/RXR peaks were split into quartiles based on binding strength and proportion of sites overlapping murine PPARG binding regions lifted over from mm8 to hg18 was assessed. D) Changing the threshold for liftOver of mouse binding regions does not strongly influence the analysis of binding site retention. Human PPARG/RXR peaks were split into quartiles based on binding strength and proportion of sites overlapping murine PPARg binding regions lifted over from mm8 to hg18 using -minMatch 0.4 (more conservative) was assessed. E) PPARG/RXR peaks in human macrophages and PPARG peaks in murine macrophages were obtained under different significance thresholds using MACS. This analysis shows that the results are relatively robust against changes in the threshold and retention does not approach 10% even under the most conservative setup. Dark grey bar show the proportion of retained human sites. Light grey bars show the proportion of retained murine site. Comparison was done by lifting the murine sites from onto the human genome as before. F) Comparison of the proportion of human PPARG/RXR sites at which a PPARG motif was detected depending on the detection threshold. PPARG motifs were detected using FIMO (Grant et al. 2010). Increasing the detection p-Value leads to detection of more motifs. However, this also led to increased calls in random regions.
(TIF)

Figure S3 **Expression analysis of RSG-responsive genes helps to define direct target genes.** A) Association of PPARG/RXR binding sites with RSG regulated genes in human dendritic cells. Expression data obtained from Szatmari et al.. All genes that were induced by RSG in at least on of the reported time-points (6 h, 12 h and 5 d) were used. Significance was calculated using Fisher's exact test B) Proportion of RSG regulated genes among human-specific and shared targets. Expression data from THP-1 cells (this study) C) Proportion of RSG regulated genes among human-specific and shared targets. Expression data obtained from dendritic cells (Szatmari et al., 2007). D) Enrichment of biological processes among species-specific PPARG/RXR target genes in human macrophages. Shown are the top 10 categories identified by PANTHER. E) Enrichment of biological processes among shared (both indirectly and directly shared) PPARG/RXR target genes in human macrophages. Shown are the top 10 categories identified by PANTHER. F) Proportions of PPARG target genes from different categories (species-specific, indirectly shared and directly shared) that were differentially regulated in murine *PPARG−*/− monocytes. Expression data was obtained from Hevener et al. (2007).
(TIF)

Figure S4 **Genome-wide co-occurrence of PPARG and PU.1 in human and mouse macrophages.** A) Secondary motif identified at PPARG/RXR using cisfinder. B) Transfac motif for ETS family factors matches the identified secondary motif; Similarity calculated with STAMP C) Identified ETS motif is detected at the centre of around 40% of all PPARG/RXR binding sites (300 bp interval). D) Proportion of PPARG sites with a detectable PPARG motif in human macrophages and in two replicates from human adipocytes (ChIP-seq data from Mikkelsen et al.). Both cell types show very similar proportions of sites with PPARG motifs E) Proportion of PPARG sites with a detectable PU.1 motif in human macrophages and in two replicates from human adipocytes (ChIP-seq data from Mikkelsen et al.). While almost 40% of PPARG/RXR sites in macrophages contain a detectable PU.1 motif, just above 16% of the PPARG sites in human adipocytes contain a PU.1 motif. F) Comparison of PU.1 peaks called using CCAT (used for our analysis) and MACS show good agreement. G) Overlap of PPARG/RXR sites with PU.1 binding based on PPARG/RXR peak enrichment. PPARG/RXR peaks were split into quartiles based on binding strength and proportion of sites overlapping PU.1 binding regions is plotted for each quartile. The proportion of PPARG peaks that overlaps a PU.1 peak is shown for each quartile. H) Distribution of distances between the centers of PPARG and PU.1 peaks at PPARG/RXR that coincide with PU.1 sites. I) Comparison of PU.1 peak calls in murine macrophages. Data were obtained from Lefterova et al. and compared are the published peak calls to peaks called on the same data using MACS.
(TIF)

Figure S5 **PU.1 aided turnover model for PPARG binding sites and selected example.** A) Human PPARG/RXR sites which contained a PPARG motif both in human as well as at orthologous loci in mouse were grouped into PPARG overlap-sites and human-specific sites. Co-binding of PU.1 and presence of a PU.1 motif proportion was assessed in the mouse genome. Significance was calculated using Fisher's exact test. B) Binding profiles of PU.1 and PPARG in human (left) and mouse (right) macrophages around the indirectly shared target gene *LIPA*/*Lipa*. C) Binding profiles of PPARG and PU.1 in human (left) and mouse (right) macrophages at the human-specific PPARG binding site in *LIPA/Lipa* (R2 from B)) demonstrate the presence of the respective motifs underlie PPARG and PU.1 binding events D) PPARG binding motif found at the *Lipa* R1 locus in mouse (upper panel). Comparison with the aligned region at the human locus (lower panel) shows absence of a PPARG motif. E) PPARG binding motif found at the *LIPA* R2 locus in human (upper panel). This time comparison with the aligned region at the murine locus reveals mutations within the PPARG motif (lower panel).
(TIF)

Table S1 **PPARG/RXR binding sites and associated features.** Binding sites for PPARG were obtained using CCAT and only sites with RXR enrichment within proximity (<500 bp) were retained. 2133 PPARG/RXR sites were used for further analysis. A 150 bp interval surrounding the peak centre was used for most analysis. Shown are the coordinates in BED format (150 bp interval) (Column 1–3); Column 4: binding site ID (indicating middle of BS); Column 5: PPARG ChIP-Seq enrichment (calculated by CCAT); Column 6: significance of enrichment (calculated by CCAT); Column 7: site conserved in mouse (yes/no); Column 8: site overlapping a PhastCons element (yes/no); Column 9: PU.1 binding sites within 150 bp of PPARG/RXR sites (yes/no).
(XLSX)

Table S2 **Summary ChIP-Seq libraries.** Number of lanes and sequenced tags for all ChIP-Seq libraries are listed in this table. Raw and processed data was submitted to GEO under accession number: GSE25608.
(PDF)

Table S3 **Putative PPARg target genes.** For each binding sites the nearest gene within 100 kb is listed. Initial identification is based on the distance to the gene body, the distance to the TSS of the gene is then calculated. Shown are PPARG/RXR binding sites within 100 kb of the TSS of a RefGene.
(XLSX)

Table S4 **Conserved targets genes.** PPARG/RXR bound target genes in human macrophages (Table3) were compared to PPARG target genes in murine macrophages. Mouse PPARG binding sites from Lefterova et al. were used to identify putative target genes in mouse. Indirectly conserved and conserved PPARG/RXR target genes are listed. Genes with at least one PPARg binding sites within 100 kb of their TSS were considered target genes.
(XLSX)

Table S5 **Rosiglitazone responsive genes in THP-1 cells.** Genes identified as RSG responsive in a time course experiment in PMA-differentiated THP-1 cells. Listed are Symbols, Illumina Probe IDs and fold change of RSG treated samples compared to vehicle (DMSO) treated samples for each time point. Fold change is shown as the log2 ratio. Listed genes show a fold change >1.5 for at least one time point.
(XLSX)

Table S6 **Degrees of conservation for PPARG target genes correlates with RSG responsiveness.** Putative PPARG targets were grouped by PPARG binding in human and mouse (human-specific, indirectly shared and directly shared) and assessed for RSG responsiveness. In addition we used a set RSG responsive gene identified in human dendritic cells (Szatmari et al., 2007) to increase the sensitivity. A total of 481 RSG responsive genes in myeloid cells were compared to PPARG.
(XLSX)

Table S7 **PU.1 binding sites in human macrophages (THP-1).** PU.1 binding sites determined with CCAT. For all significant sites a 150 bp interval around the centre of the middle is listed along with the Binding ID, enrichment and significance. The last column indicates if the PU.1 binding site is conserved between human and mouse macrophages.
(XLSX)
