# Supplementary figures and images for: PPARG Binding Landscapes in Macrophages Suggest a Genome-Wide Contribution of PU.1 to Divergent PPARG Binding in Human and Mouse
Source: PLoS One. 2012 Oct 31;7(10):e48102. doi: 10.1371/journal.pone.0048102 (PMC3485280; doi:10.1371/journal.pone.0048102)

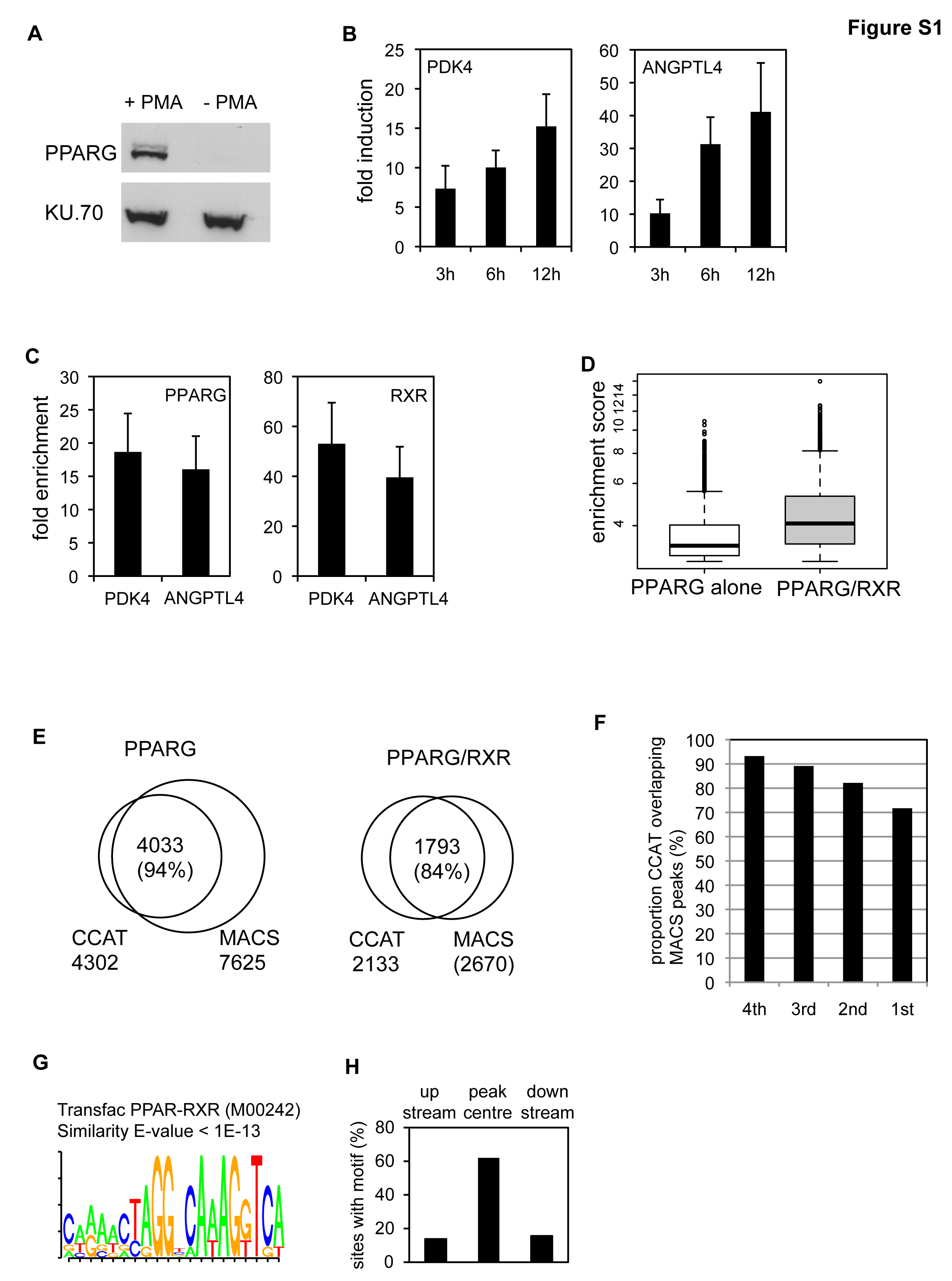

Supplement: Figure S1 — PPARG ChIP-seq reliably detects PPARG binding sites in THP-1 cells. A) Western blot for PPARG after and before PMA differentiation of THP-1 cells. KU70 was used as control. B) Induction of mRNA levels of PDK4 and ANGPTL4 in PMA differentiated THP-1 cells after RSG treatment. Shown is the fold induction compared to vehicle (DMSO) treated cells. RNA was harvested at indicated timepoints C) ChIP enrichment detected at PPARG binding sites adjacent to PDK4 and ANGPTL4 using antibodies against PPARG and RXR D) enrichment score determined by CCAT for PPARG peaks at PPARG binding sites without and with RXR support E) Comparison of peaks obtained with different peak calling algorithms. Venn-diagrams showing overlap between peaks called with CCAT and MACS, respectively. Left diagram shows PPARG peaks and PPARG/RXR peaks are compared to the right. F) Overlap between CCAT and MACS calls for PPARG/RXR peaks for quartiles of PPARG/RXR binding sites. Quartiles are based on CCAT enrichment score. The proportion of PPARG/RXR peaks called with CCAT that overlaps a PPARG/RXR peak called with MACS is shown for each quartile G) Transfac motif matching the identified PPARG/RXR binding motif; Similarity calculated with STAMP H) Identified PPARG/RXR motif is detected at the majority of binding sites and located at the centre of the peak (300 bp interval). (TIF) [file pone.0048102.s001.tif]

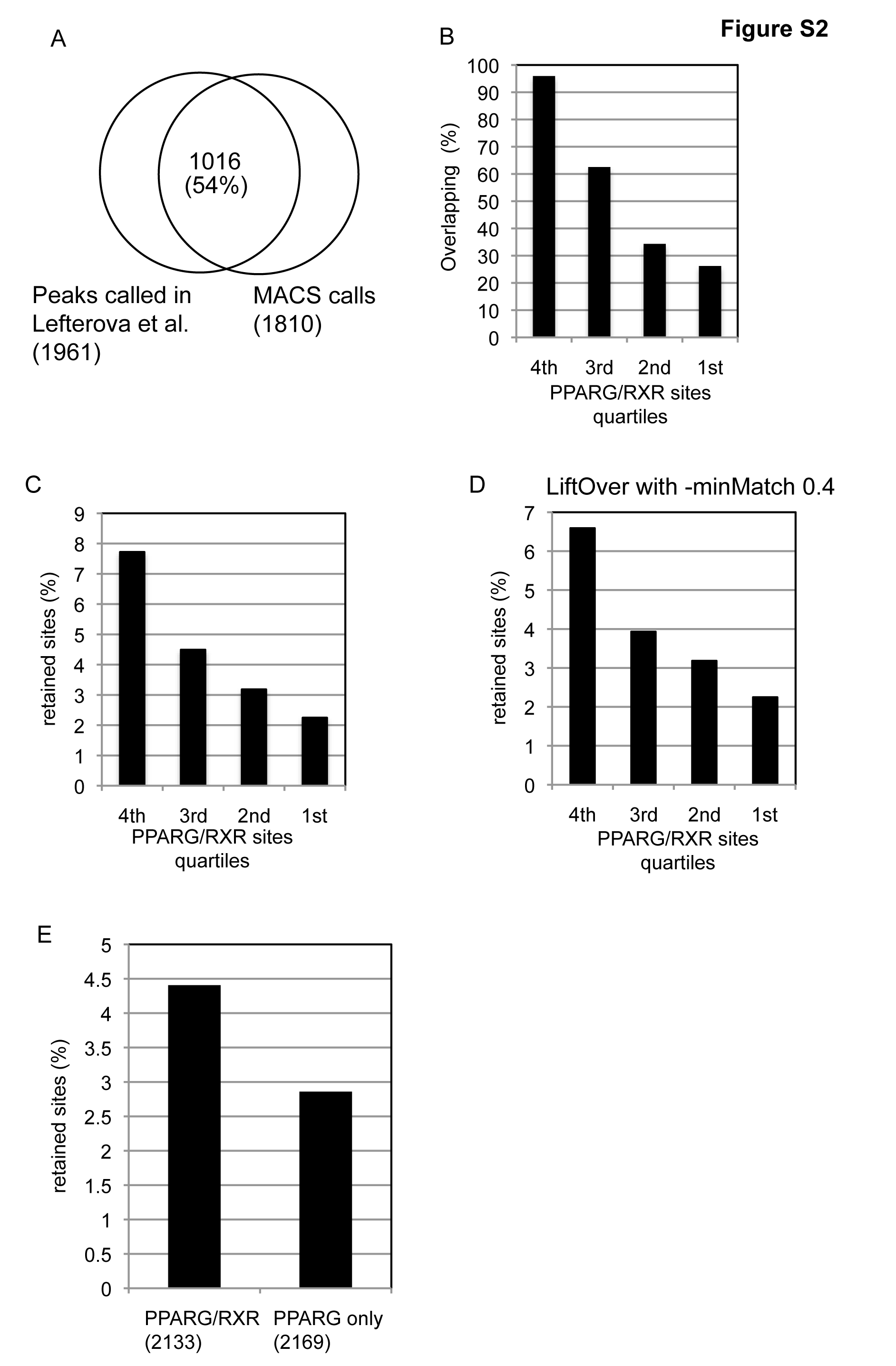

Supplement: Figure S2 — Low binding site retention is robust to different peak calling thresholds. A) Comparison of PPARG peaks in murine macrophages reported by Lefterova et al. and peaks called from the same raw data using MACS. B) Overlap between original peaks published by Lefterova et al. and MACS calls for PPARG. Peaks called by Lefterova et al. were split into quartiles based on reported peaks scores. The proportion of PPARG peaks in the original dataset that overlaps a PPARG peak called by MACS is shown for each quartile. C) Binding site retention is biased towards strong PPARG/RXR peaks. Human PPARG/RXR peaks were split into quartiles based on binding strength and proportion of sites overlapping murine PPARG binding regions lifted over from mm8 to hg18 was assessed. D) Changing the threshold for liftOver of mouse binding regions does not strongly influence the analysis of binding site retention. Human PPARG/RXR peaks were split into quartiles based on binding strength and proportion of sites overlapping murine PPARg binding regions lifted over from mm8 to hg18 using -minMatch 0.4 (more conservative) was assessed. E) PPARG/RXR peaks in human macrophages and PPARG peaks in murine macrophages were obtained under different significance thresholds using MACS. This analysis shows that the results are relatively robust against changes in the threshold and retention does not approach 10% even under the most conservative setup. Dark grey bar show the proportion of retained human sites. Light grey bars show the proportion of retained murine site. Comparison was done by lifting the murine sites from onto the human genome as before. F) Comparison of the proportion of human PPARG/RXR sites at which a PPARG motif was detected depending on the detection threshold. PPARG motifs were detected using FIMO (Grant et al. 2010). Increasing the detection p-Value leads to detection of more motifs. However, this also led to increased calls in random regions. (TIF) [file pone.0048102.s002.tif]

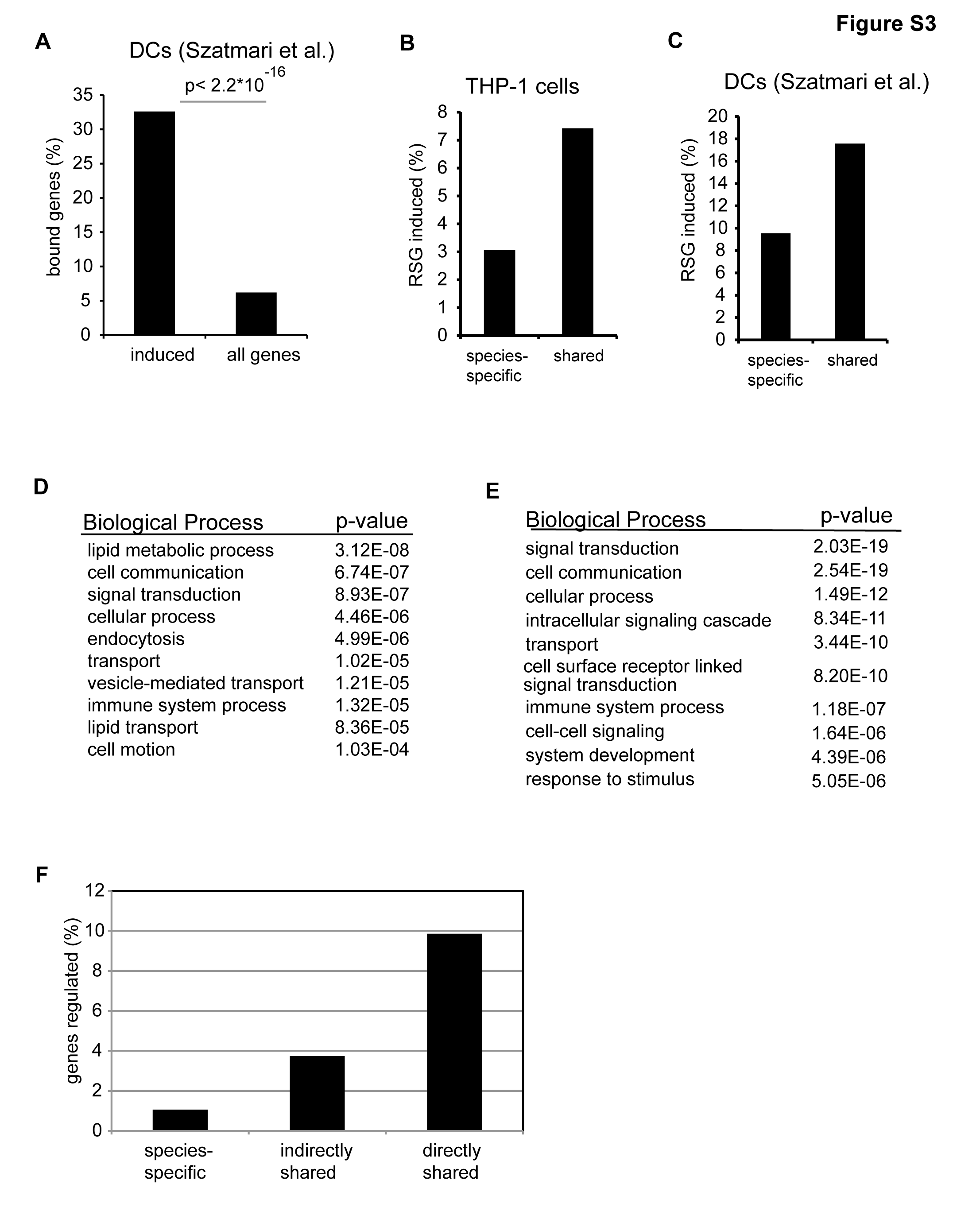

Supplement: Figure S3 — Expression analysis of RSG-responsive genes helps to define direct target genes. A) Association of PPARG/RXR binding sites with RSG regulated genes in human dendritic cells. Expression data obtained from Szatmari et al.. All genes that were induced by RSG in at least on of the reported time-points (6 h, 12 h and 5 d) were used. Significance was calculated using Fisher's exact test B) Proportion of RSG regulated genes among human-specific and shared targets. Expression data from THP-1 cells (this study) C) Proportion of RSG regulated genes among human-specific and shared targets. Expression data obtained from dendritic cells (Szatmari et al., 2007). D) Enrichment of biological processes among species-specific PPARG/RXR target genes in human macrophages. Shown are the top 10 categories identified by PANTHER. E) Enrichment of biological processes among shared (both indirectly and directly shared) PPARG/RXR target genes in human macrophages. Shown are the top 10 categories identified by PANTHER. F) Proportions of PPARG target genes from different categories (species-specific, indirectly shared and directly shared) that were differentially regulated in murine PPARG−/− monocytes. Expression data was obtained from Hevener et al. (2007). (TIF) [file pone.0048102.s003.tif]

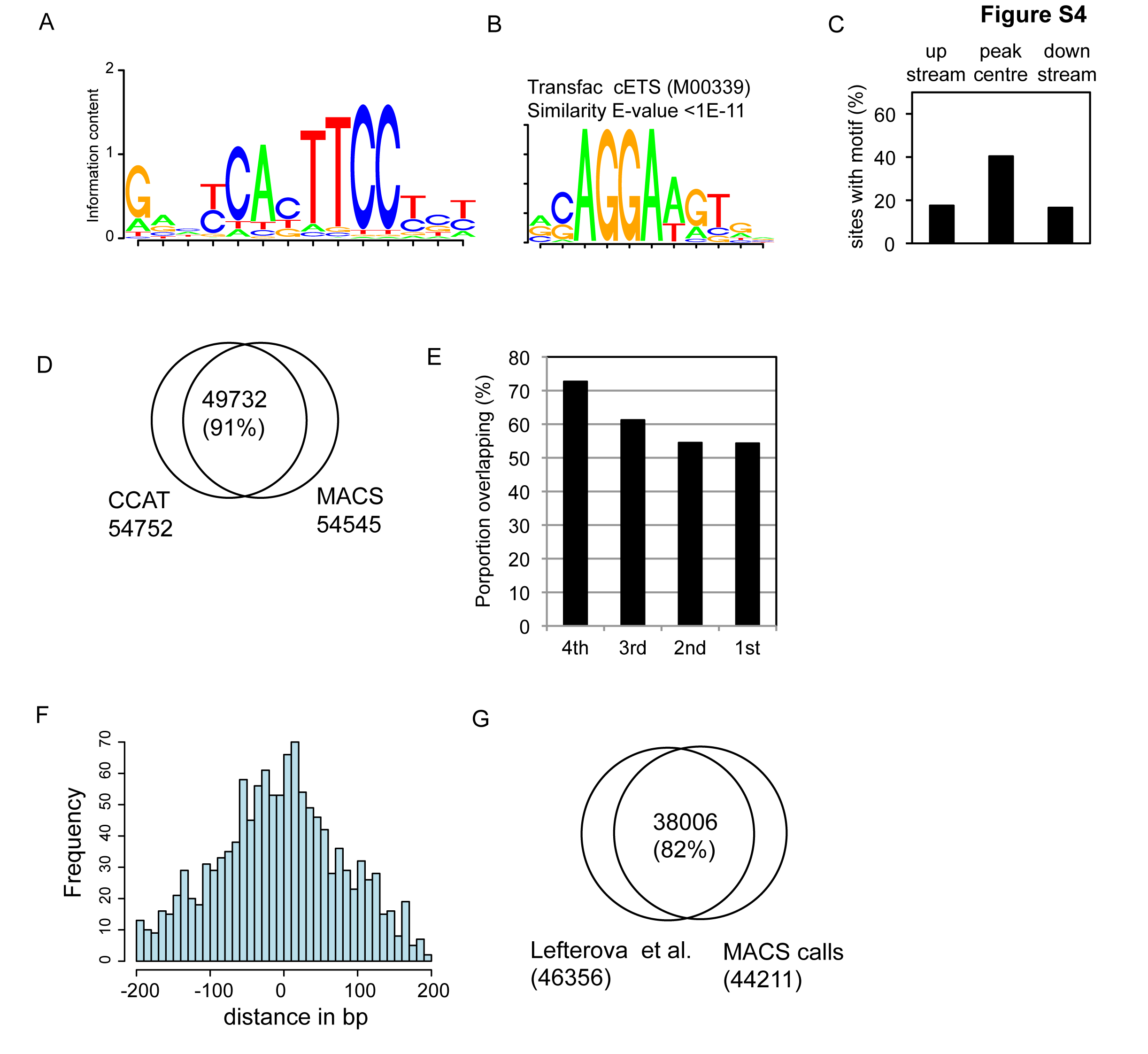

Supplement: Figure S4 — Genome-wide co-occurrence of PPARG and PU.1 in human and mouse macrophages. A) Secondary motif identified at PPARG/RXR using cisfinder. B) Transfac motif for ETS family factors matches the identified secondary motif; Similarity calculated with STAMP C) Identified ETS motif is detected at the centre of around 40% of all PPARG/RXR binding sites (300 bp interval). D) Proportion of PPARG sites with a detectable PPARG motif in human macrophages and in two replicates from human adipocytes (ChIP-seq data from Mikkelsen et al.). Both cell types show very similar proportions of sites with PPARG motifs E) Proportion of PPARG sites with a detectable PU.1 motif in human macrophages and in two replicates from human adipocytes (ChIP-seq data from Mikkelsen et al.). While almost 40% of PPARG/RXR sites in macrophages contain a detectable PU.1 motif, just above 16% of the PPARG sites in human adipocytes contain a PU.1 motif. F) Comparison of PU.1 peaks called using CCAT (used for our analysis) and MACS show good agreement. G) Overlap of PPARG/RXR sites with PU.1 binding based on PPARG/RXR peak enrichment. PPARG/RXR peaks were split into quartiles based on binding strength and proportion of sites overlapping PU.1 binding regions is plotted for each quartile. The proportion of PPARG peaks that overlaps a PU.1 peak is shown for each quartile. H) Distribution of distances between the centers of PPARG and PU.1 peaks at PPARG/RXR that coincide with PU.1 sites. I) Comparison of PU.1 peak calls in murine macrophages. Data were obtained from Lefterova et al. and compared are the published peak calls to peaks called on the same data using MACS. (TIF) [file pone.0048102.s004.tif]

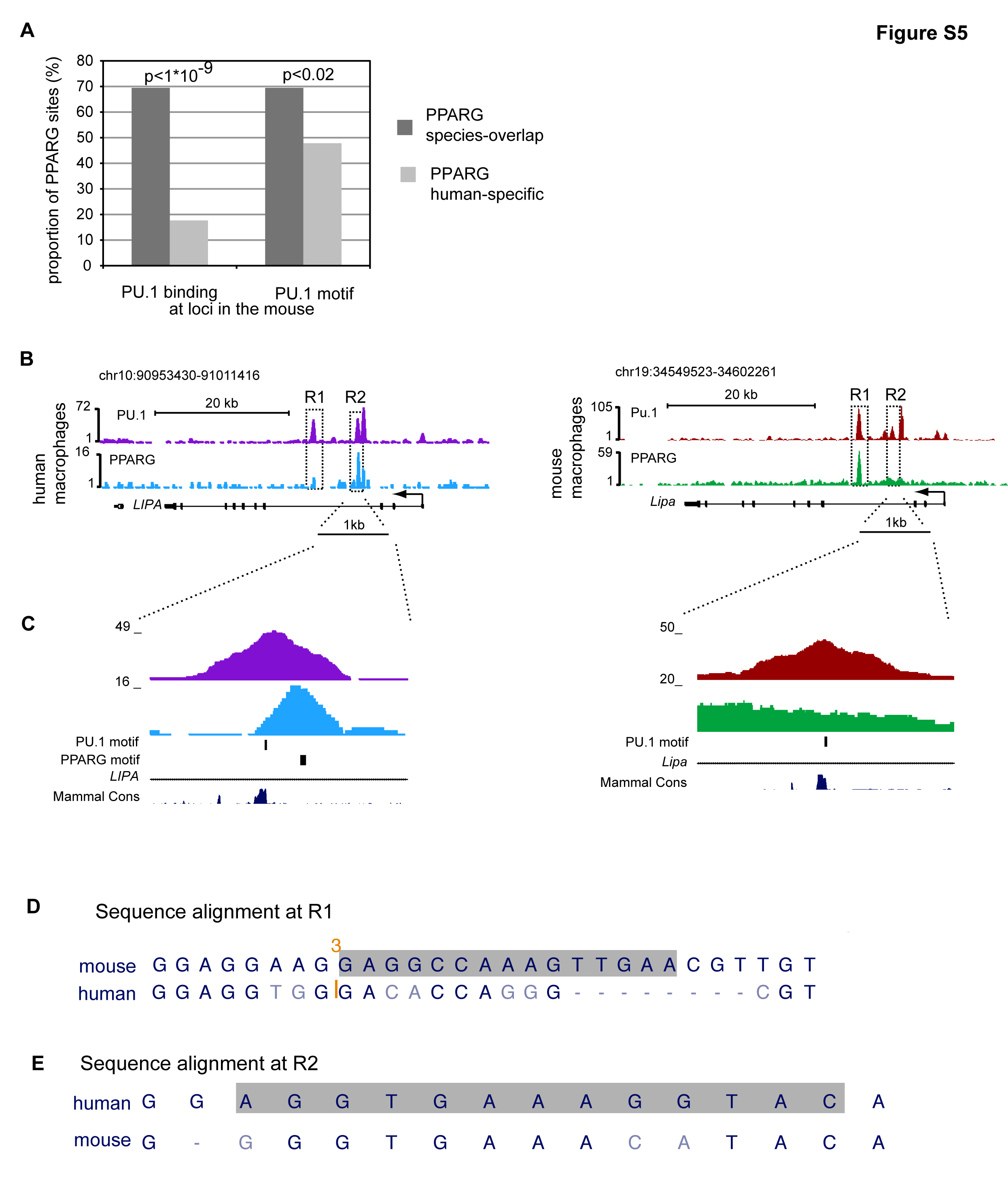

Supplement: Figure S5 — PU.1 aided turnover model for PPARG binding sites and selected example. A) Human PPARG/RXR sites which contained a PPARG motif both in human as well as at orthologous loci in mouse were grouped into PPARG overlap-sites and human-specific sites. Co-binding of PU.1 and presence of a PU.1 motif proportion was assessed in the mouse genome. Significance was calculated using Fisher's exact test. B) Binding profiles of PU.1 and PPARG in human (left) and mouse (right) macrophages around the indirectly shared target gene LIPA/Lipa. C) Binding profiles of PPARG and PU.1 in human (left) and mouse (right) macrophages at the human-specific PPARG binding site in LIPA/Lipa (R2 from B)) demonstrate the presence of the respective motifs underlie PPARG and PU.1 binding events D) PPARG binding motif found at the Lipa R1 locus in mouse (upper panel). Comparison with the aligned region at the human locus (lower panel) shows absence of a PPARG motif. E) PPARG binding motif found at the LIPA R2 locus in human (upper panel). This time comparison with the aligned region at the murine locus reveals mutations within the PPARG motif (lower panel). (TIF) [file pone.0048102.s005.tif]
