# Supplementary material for: PPARG Binding Landscapes in Macrophages Suggest a Genome-Wide Contribution of PU.1 to Divergent PPARG Binding in Human and Mouse
Source: PLoS One. 2012 Oct 31;7(10):e48102. doi: 10.1371/journal.pone.0048102 (PMC3485280; doi:10.1371/journal.pone.0048102)
Supplement: Table S2 — Summary ChIP-Seq libraries. Number of lanes and sequenced tags for all ChIP-Seq libraries are listed in this table. Raw and processed data was submitted to GEO under accession number: GSE25608. (PDF) [file pone.0048102.s007.pdf]

Library	# lanes	# sequenced tags
PPARg	3	10232113
RXR	1	12132706
IgG	1	13687635
PU.1	1	13429572

Data are deposited at GEO as SuperSeries under the accession number GSE25608
